# Elastomeric Core‐Conductive Shell Slurry Additive Stabilizes High‐Voltage NCM811 Cathodes via Mechanical‐Electrical Dual Regulation

**DOI:** 10.1002/advs.77552

**Published:** 2026-09-01

**Authors:** Jiamin Duan, Xingchen Liu, Jiapei Li, Zhuijun Xu, Said Amzil, Baohu Wu, Yiyao Xiao, Wenjun Zhang, Tong Yu, Xiaohong Li, Wenwen Xue, Xiangyu Chen, Changwei Liu, Jie Gao, Peter Müller‐Buschbaum, Ya‐Jun Cheng, Yonggao Xia

**Affiliations:** ^1^ Ningbo Institute of Materials Technology & Engineering Chinese Academy of Sciences Ningbo Zhejiang People's Republic of China; ^2^ Center of Materials Science and Optoelectronics Engineering University of Chinese Academy of Sciences Beijing People's Republic of China; ^3^ Shanghai Institute of Space Power‐Sources Shanghai People's Republic of China; ^4^ Department of Materials Science and Engineering & Center of Super‐Diamond and Advanced Films (COSDAF) City University of Hong Kong Kowloon Hong Kong SAR People's Republic of China; ^5^ TUM School of Natural Sciences Department of Physics Chair For Functional Materials Technical University of Munich Garching Germany; ^6^ Jülich Centre for Neutron Science (JCNS) At Heinz Maier‐Leibnitz Zentrum (MLZ) Forschungszentrum Jülich Garching Germany; ^7^ College of Chemistry Chemical Engineering and Materials Science Soochow University Suzhou Jiangsu People's Republic of China; ^8^ College of Renewable Energy Hohai University Changzhou Jiangsu People's Republic of China; ^9^ Easpring (Changzhou) New Material Co., Ltd. Changzhou Jiangsu People's Republic of China

**Keywords:** artificial cathode‐electrolyte interface, core‐shell structure, high voltage, nickel‐rich layered cathode, slurry additive

## Abstract

High‐voltage (>4.3 V) Ni‐rich layered cathodes are central to high‐energy‐density lithium‐ion batteries, yet their practical operation is limited by coupled mechanical degradation, sluggish interfacial charge transport, and parasitic cathode‐electrolyte reactions. Herein, we report a slurry‐compatible strategy to construct a phase‐separated mosaic‐like interphase on LiNi_0.8_Co_0.1_Mn_0.1_O_2_ (NCM811) cathodes using poly(acrylonitrile‐styrene‐acrylate) (ASA) as a multifunctional additive. During N‐methyl‐2‐pyrrolidone (NMP)‐based slurry casting and electrode drying process, the core‐shell ASA precursor undergoes structural reorganization, forming an ASA‐derived interphase composed of poly (butyl acrylate) (PBA)‐rich elastomeric regions and poly(styrene‐co‐acrylonitrile) (SAN)‐rich polar/aromatic regions. The PBA‐rich component buffers volume‐change‐induced stress and suppresses particle cracking, while the SAN‐rich component contributes to Li^+^‐related interfacial affinity and enhances electron conductive. This phase‐separated, mosaic‐like interphase promotes stress dissipation from intercalation (deintercalation), enabling simultaneous regulation of mechanical integrity, interfacial chemistry, and electrode kinetics. Consequently, the optimized cathode exhibits reduced polarization, enhanced Li^+^ diffusion, suppressed transition‐metal dissolution, and stabilized electrode interphases. In practical NCM811||graphite pouch cells under 4.5 V operation, capacity retention increases from 55.2% to 78.7% after 500 cycles, increased by 43%. This strategy highlights the importance of regulating the phase organization of polymeric slurry additives, offering a practically compatible route for stabilizing high‐voltage layered oxide cathodes.

## Graphical abstract

A core‐shell ASA slurry additive constructs a mosaic‐like interface on high‐voltage NCM811 cathodes. This architecture decouples interfacial functions: the elastomeric core buffers mechanical stress to prevent microcracking, while the conductive shell facilitates rapid ion/electron transport, enabling mechanical‐electrical dual regulation and enhanced cycling stability in 4.5 V pouch cells.

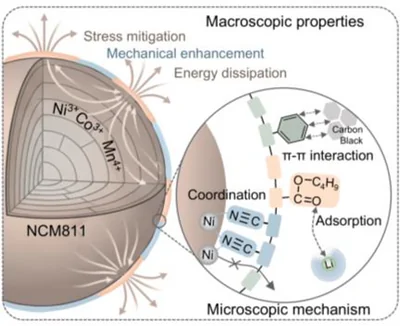

## 1 Introduction

High‐voltage operation (>4.3 V) of LiNi_0.8_Co_0.1_Mn_0.1_O_2_ (NCM811) cathodes is essential to achieve >300 Wh kg^−1^ energy density targets for electric vehicles [1, 2, 3, 4]. However, NCM811 suffers from capacity degradation during high‐voltage cycling, limiting its widespread commercial adoption [5]. At highly delithiated states, extensive lithium extraction induces anisotropic volume changes that trigger severe intragranular microcracking [6, 7]. These exposed surfaces undergo parasitic oxidation, leading to the formation of an unstable cathode‐electrolyte interphase (CEI) and dissolving transition metals (Ni^2+^, Mn^2+^) into the electrolyte. The dissolved species can then migrate to the anode, where they further catalyze continuous solid‐electrolyte interphase (SEI) growth and increase cell impedance. Conventional poly (vinylidene fluoride) (PVDF) binders tend to exacerbate this degradation, as they primarily provide passive mechanical adhesion without chemical functionality to suppress transition metal dissolution, stabilize the CEI, and reinforce particles against fracture over repeated cycles.

Despite considerable efforts to construct stable CEI layers using electrolyte additives or surface coatings, practical challenges remain [8]. Electrolyte additive‐mediated CEI formation often complicates the electrolyte composition and requires stringent operational protocols [9, 10], while surface coating techniques frequently yield inhomogeneous layers with compromised lithium‐ion conductivity [11, 12]. In contrast, integrating functional polymeric additives into the cathode slurry offers a scalable and practical route to regulate the cathode surface during electrode fabrication without modifying the bulk material [13, 14].

Previous polymeric slurry additives have demonstrated that polar groups, elastomeric segments, or ion‐interacting moieties can stabilize cathode interfaces by coordinating surface transition‐metal species, suppressing electrolyte decomposition, or constructing artificial CEI layers [15, 16, 17]. However, most reported systems mainly focus on chemical functionality, while the spatial organization of different functional components within the polymer‐derived interphase has received less attention. A homogeneous or overly dense polymer layer may improve interfacial stability but can also limit electrolyte accessibility, Li^+^ transport, or electronic conductivity. Therefore, designing polymeric additives that can spatially partition mechanical protection, interfacial coordination, and charge‐transport regulation remains an important challenge for high‐voltage Ni‐rich cathodes.

**FIGURE 1:**
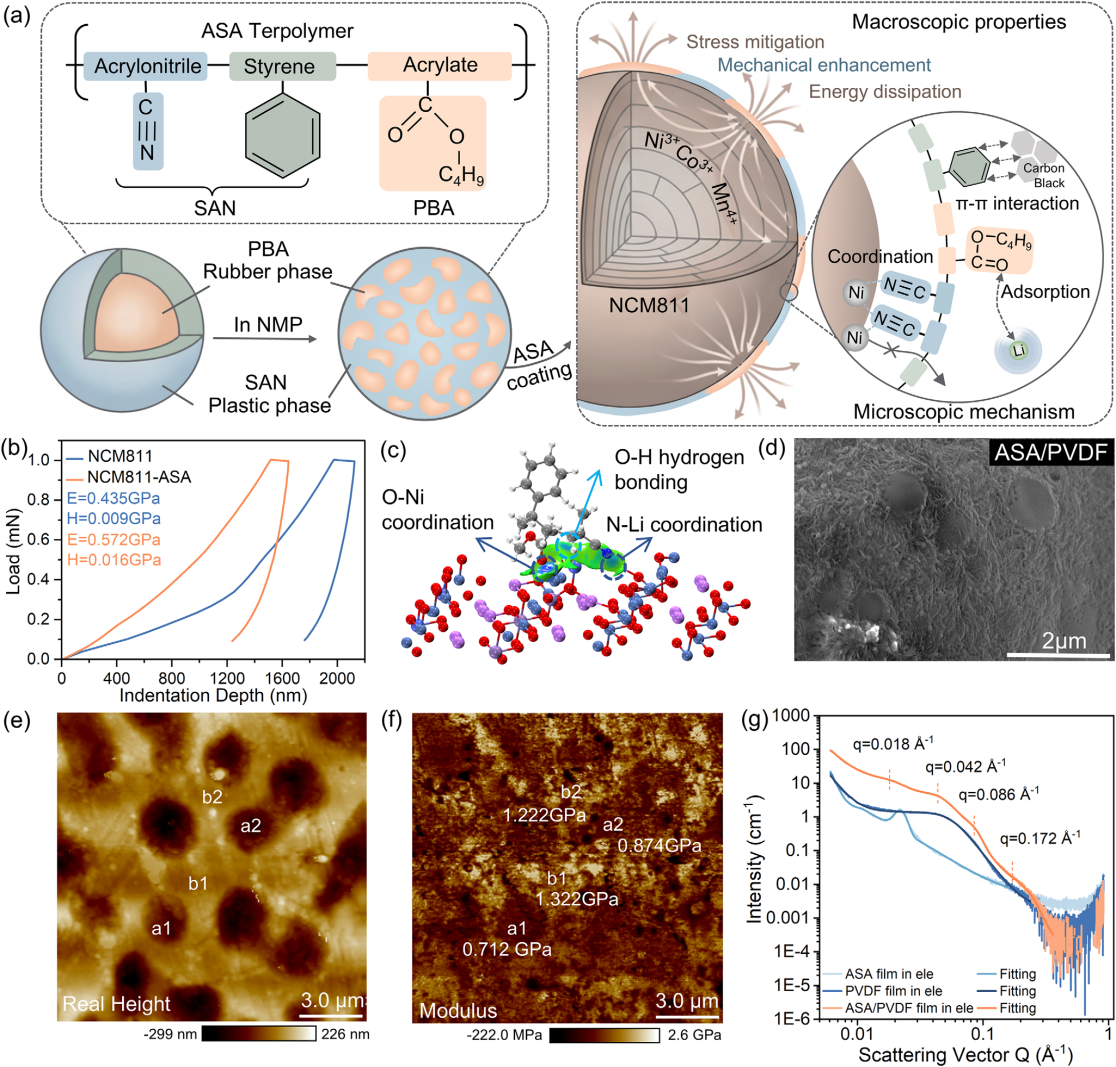
(a) Schematic illustration of the proposed evolution of the core‐shell ASA precursor into an ASA‐derived mosaic‐like interphase on NCM811, together with its proposed mechanical and chemical roles. (b) Nanoindentation load‐displacement curves of pristine NCM811 and NCM811‐ASA electrodes. (c) mIGM analysis of the interfacial interactions between an ASA fragment and the NCM811 surface. (d) SEM image of the ASA/PVDF coating on the NCM811 electrode surface. AFM nanomechanical mapping of the ASA film: (e) height image and (f) corresponding modulus map. (g) SAXS profiles and corresponding fits of ASA, PVDF, and ASA/PVDF films after electrolyte swelling.

In this work, Poly(acrylonitrile‐styrene‐acrylate) (ASA) is introduced as a phase‐separating polymeric slurry additive to construct a mosaic‐like interphase on NCM811 cathodes. Pristine ASA possesses a core‐shell precursor structure, where the core is composed of cross‐linked poly(butyl acrylate) (PBA) elastomer, and the shell is poly(styrene‐acrylonitrile) (SAN) copolymer containing polar nitrile groups and aromatic styrene units (Figure 1a) [18, 19, 20]. The inherent immiscibility between PBA and SAN endows this core‐shell precursor with two thermodynamically distinct polymer fractions [21]. During N‐methyl‐2‐pyrrolidone (NMP)‐based slurry casting and electrode drying process, the ASA additive undergoes spontaneous phase separation and solvent evaporation‐driven structural rearrangement, which converts this precursor architecture into a phase‐separated ASA‐derived interphase. This controllable phase separation of the polymeric additives delivers a viable strategy to balance interfacial stability and electrochemical kinetics for high‐voltage Ni‐rich cathode materials.

The resulting mosaic‐like interphase enables spatial functional partitioning at the cathode surface. The PBA‐rich elastomeric regions accommodate volume‐change‐induced stress and suppress crack propagation during high‐voltage cycling, thereby improving the mechanical robustness of the cathode interface. Meanwhile, the SAN‐rich regions provide dual interfacial functions through their styrene and acrylonitrile units: the aromatic styrene units interact with conductive carbon through π‐π interactions [22, 23], while the polar nitrile groups from acrylonitrile units can coordinate with surface transition‐metal species, helping anchor the ASA‐derived interphase onto NCM811 particles [24]. Such spatial separation helps decouple mechanical buffering from charge‐transport regulation, reducing the kinetic penalty commonly associated with dense polymer‐derived protective layers. As a result, the ASA‐derived interphase simultaneously stabilizes the cathode surface, preserves electrolyte accessibility, and suppresses cathode‐to‐anode degradation in high‐voltage NCM811||graphite cells.

## 2 Results and Discussion

### 2.1 Mechanism of ASA‐Coating NCM811

ASA was first dissolved in NMP, mixed with NCM811 particles, and subsequently dried to obtain NCM811‐ASA particles. Scanning electron microscopy (SEM) was then used to compare the surface morphologies of pristine NCM811 and NCM811‐ASA particles (Figure S1). Compared with the relatively clean surface of pristine NCM811, NCM811‐ASA exhibits a thin polymer‐derived layer covering the particle surface, indicating the deposition of ASA on NCM811. The presence of ASA‐derived functional groups on the modified NCM811 surface was further verified by Fourier‐transform infrared (FTIR) spectroscopy and X‐ray photoelectron spectroscopy (XPS). Compared with pristine NCM811‐Powder, NCM811‐ASA‐Powder shows characteristic ASA‐related signals in the FTIR spectrum, including the ester C═O stretching vibration at 1730 cm^−1^ and the styrene‐related aromatic C‐H out‐of‐plane bending vibration at 698 cm^−1^ (Figure S2) [25, 26, 27]. In the C 1s XPS spectra, the main peaks are assigned to C─C/C─H (284.4 eV), C─O/C≡N (286.3 eV), C═O (288.5 eV), and CO_3_^2−^ (289.7 eV) (Figure S3a) [28]. Compared with NCM811‐Powder, NCM811‐ASA‐Powder exhibits enhanced C─O/C≡N and C═O contributions, consistent with the introduction of ASA‐derived polar functional groups. In the N 1s spectra (Figure S3b), no obvious nitrogen signal is observed for NCM811‐Powder, whereas NCM811‐ASA‐Powder exhibits a distinct N 1s peak at 399.3 eV, which can be assigned to nitrile‐related nitrogen from acrylonitrile units. These observations support the formation of an ASA‐derived coating layer on the NCM811 particle surface.

The mechanical impact of the ASA slurry additive on NCM811 electrodes was evaluated by nano‐indentation measurements. Here, NCM811 refers to the pristine electrode prepared without any additive, whereas NCM811‐ASA denotes the electrode prepared with an ASA‐containing slurry. Figure 1b shows that the pristine NCM811 electrode exhibits a Young's modulus of 0.435 GPa with a hardness of 0.009 GPa, whereas the NCM811‐ASA electrode exhibits enhanced mechanical properties, with the modulus increasing to 0.572 GPa and the hardness rising to 0.016 GPa. The increased Young's modulus indicates enhanced local stiffness, while the increased hardness demonstrates improved resistance to localized indentation‐induced deformation. These results suggest that the incorporation of ASA reinforces the local mechanical response of the NCM811 electrode, which may improve its ability to withstand the mechanical stresses generated during repeated high‐voltage cycling.

Following the improved local mechanical response of the NCM811‐ASA electrode, theoretical calculations were employed to elucidate the molecular interactions that stabilize ASA on the NCM811 surface. As shown in Figures S4a–f, different electrostatic potentials of ASA fragments with different monomer sequences were studied using electrostatic potential (ESP). The carbonyl O and nitrile N atoms exhibit pronounced negative ESP regions owing to the polarized C═O and C≡N groups, identifying them as potential interaction sites for positively charged species on the NCM811 surface, including Li^+^ and transition‐metal ions. Variations in monomer sequence have a negligible effect on the overall ESP distribution; therefore, a representative ASA configuration was selected for the subsequent modified independent gradient model (mIGM) analysis [29]. The resulting interaction map (Figure 1c and Figure S5) visualizes the noncovalent interactions between the ASA fragment and the NCM811 surface. The blue/cyan regions around the O─Ni and N─Li indicate attractive coordination interactions, while the O─H contact reflects a hydrogen‐bonding interaction. In addition, broad green isosurfaces between the ASA fragment and the oxide surface indicate weak van der Waals interactions. Together, these interactions provide a molecular‐level explanation for the stable attachment of ASA to NCM811 during slurry processing and subsequent drying.

To examine how solvent processing affects the internal phase structure of ASA, differential scanning calorimetry (DSC) measurements were performed on pristine core‐shell ASA powder and an ASA film obtained after NMP treatment and drying (Figure S6). Both samples exhibit two distinct glass‐transition events at approximately −45°C and 105°C, corresponding to the PBA‐rich elastomeric phase and the SAN‐rich rigid phase, respectively [27]. The retention of these two thermal transitions after NMP treatment indicates that the PBA‐rich and SAN‐rich phases preserve their distinct segmental dynamics and do not become fully homogenized during solvent treatment and subsequent drying.

To visualize how the biphasic character retained after NMP treatment manifests spatially on the cathode surface, model coating layers of ASA, ASA/PVDF, and pure PVDF were cast directly onto the NCM811 electrode surface by NMP evaporation, and their surface morphologies were examined by SEM (Figure 1d and Figure S7). The pure PVDF film forms a fiber‐rich network covering the NCM811 surface, whereas the ASA film displays discrete, circular domains that collectively produce a mosaic‐like morphology. In the ASA/PVDF film, similar circular domains remain clearly distinguishable within the fibrous PVDF matrix. Based on comparison with the individual ASA and PVDF films, these domains are attributed primarily to the ASA component, supporting the persistence of its phase‐separation tendency in the presence of PVDF and on the rough NCM811 electrode surface. To determine whether this morphology is specific to a particular film‐forming condition, ASA films were further prepared on different substrates and under different NMP evaporation conditions (Figure S8). The corresponding SEM images show that the mosaic‐like morphology persists across these conditions, although the size of the phase‐separated domains varies with the substrate and NMP evaporation conditions. The consistent existence of this morphology indicates that the mosaic‐like structure arises primarily from the intrinsic phase‐separation tendency associated with the biphasic structure of ASA, while the protocols such as the substrate feature and NMP evaporation conditions only influence the details of the mosaic structures.

Because the mosaic‐like morphology persists under different film‐forming conditions, a pure ASA film prepared by NMP evaporation was selected for Atomic force microscopy (AFM) analysis to avoid interference from other electrode components. Height and modulus maps were acquired from the same area to determine whether the different mosaic‐like regions exhibit distinct mechanical properties (Figure 1e,f). The depressed regions (a) exhibit lower modulus values of 0.712 and 0.874 GPa, whereas the surrounding continuous regions (b) exhibit higher values of 1.322 and 1.222 GPa. The close correspondence between the height and modulus contrasts confirms that the morphologically distinct regions possess different local mechanical characteristics. In conjunction with the two glass‐transition events identified by DSC, the low‐modulus depressed regions are reasonably assigned to PBA‐rich elastomeric domains, whereas the high‐modulus continuous regions are assigned to the SAN‐rich rigid phase [30]. This assignment is further supported by the force‐deformation curves (Figure S9), in which the PBA‐rich domains exhibit larger deformation than the SAN‐rich domains, confirming the mechanical contrast between the two phases. These results demonstrate that the mosaic‐like morphology reflects the coexistence of mechanically distinct PBA‐rich and SAN‐rich domains arising from phase separation. The coexistence of compliant and rigid domains may facilitate local stress accommodation while maintaining mechanical support, consistent with the increased modulus and hardness of the NCM811‐ASA electrode revealed by nanoindentation.

To investigate the phase‐separation behavior of ASA by small‐angle X‐ray scattering (SAXS), measurements were performed under different processing conditions, with experimental details provided in the Supporting Information. As shown in Figure S10a, the SAXS profile of the ASA powder exhibits a pronounced peak at q = 0.133 Å^−1^, indicating the presence of a well‐defined phase‐separated nanostructure. The characteristic domain spacing is estimated to be ≈ 4.7 nm, calculated using d = 2π/q. A related scattering feature remains detectable after NMP treatment, indicating that the nanoscale heterogeneity of ASA is retained during solvent processing. Although the peak becomes weaker in the ASA/PVDF‐NMP system because of the reduced ASA content and scattering contrast, it remains discernible after blending with PVDF. As shown in Figure S10b, the SAXS profiles of the ASA, PVDF, and ASA/PVDF films further reveal the structural features of the solid‐state systems. The ASA film retains a similar scattering feature, indicating that its nanostructured domains are preserved after film formation. Pure PVDF displays a broad peak at q ≈ 0.075 Å^−1^, reflecting the characteristic polymer matrix structure. The ASA/PVDF composite film shows a broad scattering response over q = 0.015‐0.3 Å^−1^, indicating the coexistence of structural correlations over a relatively wide nanoscale range rather than a single uniform characteristic dimension. To evaluate the structural response in a realistic battery environment, the films were further characterized after immersion in electrolyte (Figure 1g). The SAXS curves of ASA film in electrolyte and PVDF film in electrolyte exhibit a shift of the corresponding peaks to lower q values. This corresponds to an increase in the apparent domain spacing, implying that the commercial electrolyte induces polymer‐chain rearrangement and aggregation in both ASA and PVDF films. The corresponding Rg values obtained from the fits of the ASA, PVDF, and ASA/PVDF films are 48.4, 28.3, and 17.5 nm, respectively, revealing differences in their larger‐scale structural inhomogeneity. In contrast, the ASA/PVDF film in electrolyte displays multiple scattering features at q = 0.018, 0.042, 0.086, and 0.172 Å^−1^, corresponding to characteristic correlation lengths of approximately 34.9, 15.0, 7.3, and 3.7 nm, respectively. These peak positions follow an approximate integer multiple relationship, revealing the formation of an ordered phase‐separated architecture [31]. Such scattering signatures confirm a set of distinct nanoscale correlation lengths within the electrolyte‐swollen ASA/PVDF film.

Taken together, the AFM and SEM results identify mechanically distinct phase‐separated regions extending over the micrometer scale, whereas SAXS reveals structural correlations at the nanometer scale. The micrometer‐scale phase separation is attributed to the intrinsic thermodynamic immiscibility between the soft PBA and rigid SAN components [32]. The nanoscale features are tentatively attributed to local compositional fluctuations within the styrene‐acrylonitrile regions, consistent with the reported nanoscale microphase separation of covalently linked polystyrene and polyacrylonitrile blocks [33, 34]. Such hierarchical phase heterogeneity preserves the mechanically distinct polymer regions while providing nanoscale interfacial environments that may facilitate electrolyte uptake and Li^+^ transport through the polymer layer.

### 2.2 Cycling and Rate Performance

#### 2.2.1 Performance of Half‐Cells

**FIGURE 2:**
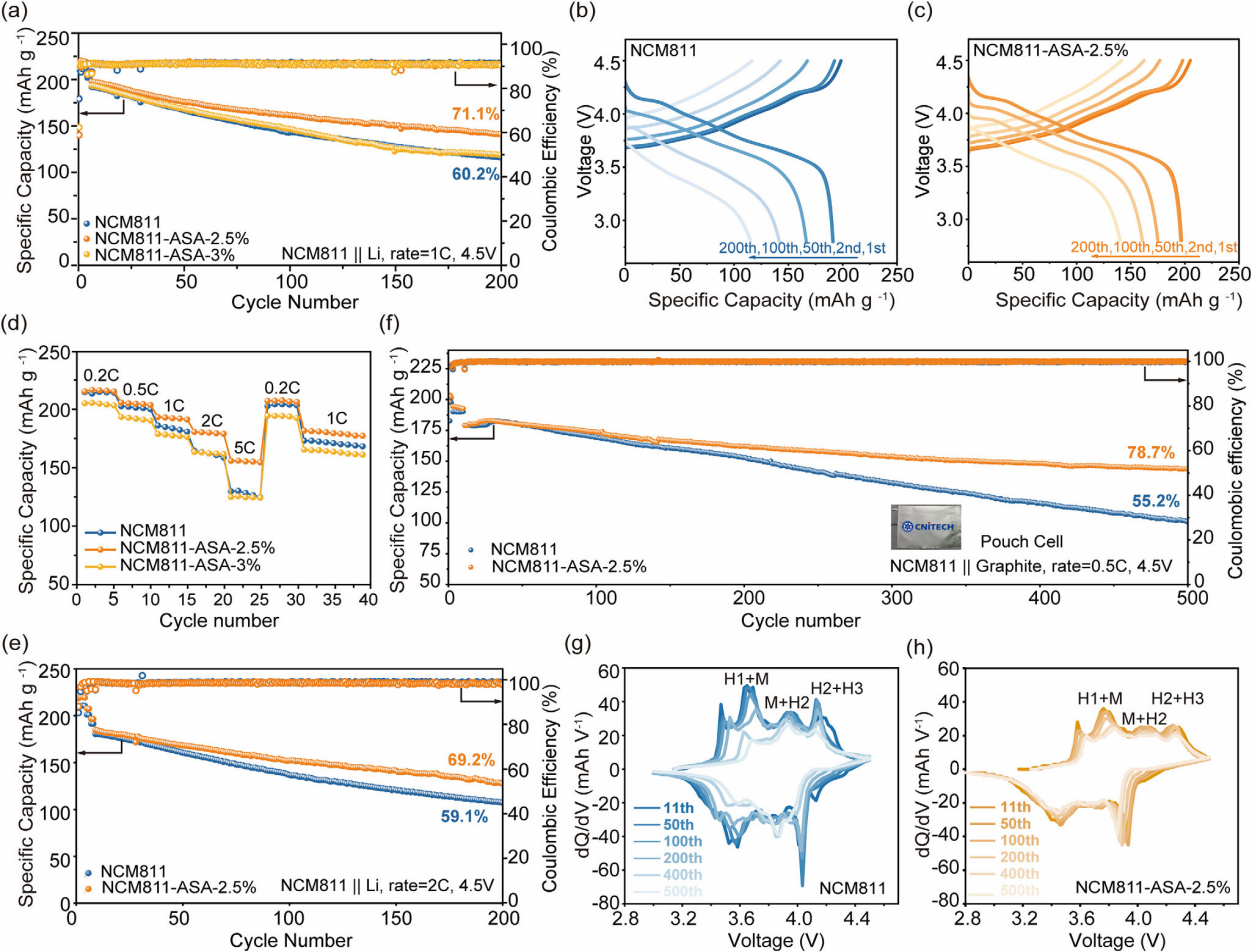
Electrochemical characterization of ASA‐modified NCM811 cathodes. (a) Cycling stability of NCM811||Li and NCM811‐ASA||Li half‐cells at 1 C. (b, c) Charge‐discharge profiles of NCM811||Li and NCM811‐ASA‐2.5%||Li half‐cells at 1C for different cycles. (d) Rate capability comparison at varying C‐rates (0.2–5 C) under 2.8–4.5 V. (e) High‐rate cycling performance at 2 C (2.8–4.5 V). (f) Pouch cell cycling performance of NCM811||graphite and NCM811‐ASA‐2.5%||graphite at 0.5 C. (g, h) The differential capacity curves (dQ/dV‐V) of (g) NCM811||graphite and (h) NCM811‐ASA‐2.5%||graphite pouch cells at 0.5 C for different cycles.

The electrochemical behavior of ASA‐modified NCM811 cathodes was systematically investigated using CR2032‐type half‐cells. As shown in Figure 2a, a comparison of the high‐voltage (4.5 V) cycling performance at 1C rate (1C = 200 mA g^−1^) shows that the cathode with 2.5% of the ASA additive exhibits the optimal cycling stability and initial coulombic efficiency. It delivers an initial specific capacity of 197 mAh g^−1^ and retains 71.1% of its capacity after 200 cycles. In contrast, the unmodified cathode exhibits a lower initial capacity (191 mAh g^−1^) and faster degradation, retaining only 60.2% of its capacity under the same conditions. Increasing the ASA content to 3% does not yield additional enhancement in capacity retention compared with the pristine NCM811 cathode, suggesting that an excessive amount of ASA does not lead to further improved cycling performance.

Figure 2b,c present the galvanostatic charge‐discharge profiles of the pristine NCM811 and ASA‐NCM811 electrodes, respectively. The NCM811‐ASA‐2.5% electrode exhibits slightly elevated charge and discharge plateaus compared to the unmodified material, together with a reduced voltage hysteresis.

Furthermore, as demonstrated in Figure 2d, the NCM811‐ASA‐2.5% electrode exhibits excellent rate performance. Specifically, the NCM811‐ASA‐2.5% electrode exhibits enhanced specific capacity compared to the unmodified NCM811, with improvements of 3 (1.0%), 7 (2.8%), 16 (7.0%), and 26 (11.7%) mAh g^−1^ at current rates of 0.5C, 1C, 2C, and 5C, respectively. However, the performance of NCM811‐ASA‐3% is not significantly improved compared to the pristine NCM811 sample. This result indicates that with an optimal amount of ASA, the electrochemical kinetics at the interface can be accelerated rather than retarded. The ASA coating layer becomes ionically conductive upon swelling by the electrolyte, which mitigates the barriers to ion transport across the ASA layer. Its styrene groups also enhance interaction with conductive carbon black, improving the electronic conductivity of the coating system. Furthermore, the strong binding capability of the carbonyl and nitrile groups of ASA facilitates lithium‐ion extraction from NCM811 particles, which is supposed to be a major factor influencing electrochemical kinetics at the cathode‐electrolyte interface. Besides, the ASA layer shields the active material from direct electrolyte contact, thereby preventing excessive electrolyte decomposition at the interface. The simultaneous improvement in cycling stability and rate capability indicates that the ASA modification does not merely act as a passive protective barrier; instead, it balances interfacial stabilization with accelerated charge transport, which is central to the proposed mechanical‐electrical dual regulation mechanism.

In view of the improved electrochemical performance by the 2.5% ASA‐coated sample at high rates, the capacity retention during long‐term cycling under high‐rate conditions was further investigated (Figure 2e). Following a stepwise activation process, the modified cathode delivers an initial capacity of 185 mAh g^−1^ at 2C, which is superior to 181 mAh g^−1^ of the original NCM811. After 200 cycles, the 2.5%‐ASA formulation maintains 128 mAh g^−1^ with 69.2% capacity retention, contrasting sharply with the unmodified cathode's 107 mAh g^−1^ (59.1% retention). This 17.1% relative capacity retention improvement at 2C underscores the critical role of ASA during high‐current operation.

#### 2.2.2 Performance of Pouch Cells

The practical implications of ASA additives are further validated through full‐cell configurations using graphite anodes. Accordingly, the impact of ASA additives on practical NCM811||graphite pouch cells was systematically evaluated. As shown in Figure 2f, both modified (NCM811‐ASA‐2.5%) and pristine cells exhibit identical initial capacities of 179 mAh g^−1^ at 0.5 C. After 500 cycles, the additive‐modified cell retains 144 mAh g^−1^ (78.7% capacity retention), significantly outperforming the unmodified cell's 100 mAh g^−1^ (55.2% retention). Figure S11a,b presents the 500th‐cycle charge‐discharge profiles of pristine and ASA‐2.5% modified NCM811 cathodes in pouch cells. The pristine electrode exhibits substantial voltage profile evolution, particularly after extended cycling, characterized by shortened voltage plateaus and increased polarization, whereas NCM811‐ASA‐2.5% maintains profiles closely matching its initial cycle, indicating superior structural stability.

Structural degradation of cathode materials, particularly through irreversible phase transitions, is a major factor contributing to capacity fade in lithium‐ion batteries. Differential capacity analysis (Figure 2g,h) reveals well‐preserved intensity and positions of H1→M→H2→H3 transition peaks in modified cells, contrasting with the attenuated and shifted peaks in pristine counterparts, providing direct evidence for suppressed irreversible phase transitions [35]. Overlaid dQ/dV profiles further demonstrate that, after 300 cycles (Figure S12), NCM811‐ASA maintains well‐defined redox features with mitigated peak broadening, while pristine NCM811 exhibits severely blurred and weakened redox responses, indicating improved preservation of high‐voltage redox reversibility. These improvements are related to the additive's dual functionality. The artificial CEI film helps maintain the structural integrity of NCM811, while coordinative interactions between polymer chains and transition‐metal species (TMs) reduce cathode dissolution [36]. Together, these effects help preserve the electrode structure and mitigate transition‐metal dissolution and structural degradation in NCM811‐ASA‐2.5%||graphite pouch cells. The pouch‐cell results demonstrate that the proposed ASA‐derived interphase remains effective under practical full‐cell conditions, where mechanical degradation, interfacial side reactions, and cathode‐anode crosstalk occur simultaneously.

### 2.3 Electrochemical Kinetics Analysis

**FIGURE 3:**
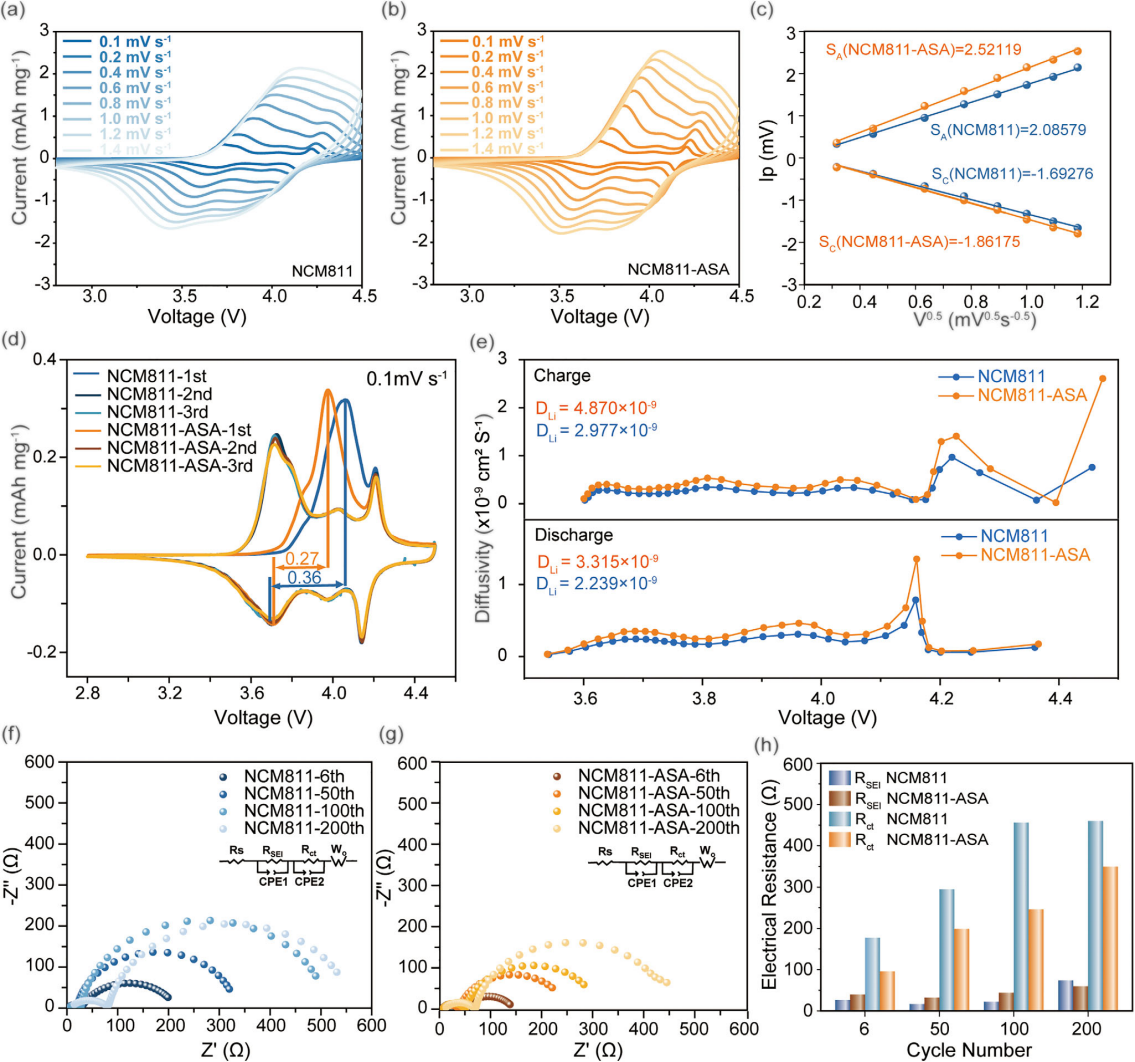
(a) CV curves of NCM811 and (b) NCM811‐ASA electrodes at scan rates from 0.1 to 1.4 mV s^−1^. (c) Linear fits of peak current versus the square root of scan rate. (d) CV profiles of NCM811 and NCM811‐ASA electrodes during the initial three cycles. (e) The diffusion coefficients D_Li+_ calculated from GITT measurements. (f) EIS spectra of NCM811 and (g) NCM811‐ASA along with the corresponding equivalent circuit models. (h) Evolution of resistance parameters (R_SEI_ and R_ct_) for NCM811 and NCM811‐ASA electrodes upon cycling.

The exceptional cycling stability motivates a detailed investigation into the electrochemical kinetics to elucidate the underlying mechanism. Figure 3a,b shows the comparative cyclic voltammetry (CV) curves for the pristine NCM811 and NCM811‐ASA electrodes across scan rates ranging from 0.1 to 1.4 mV s^−1^. Further, linear fits of the peak current as a function of the square root of the scan rate (Figure 3c) reveal significantly steeper slopes for the NCM811‐ASA compared to the pristine material, suggesting enhanced lithium‐ion diffusion coefficients (D_Li_^+^). This enhancement is related to the presence of C═O and C≡N, which facilitate faster lithium‐ion transport [37, 38].

This structural modification is further supported by the reduced potential polarization observed in Figure 3d, where the NCM811‐ASA electrode exhibits a 25% decrease in ΔE (oxidation‐reduction peak potential difference) during initial cycling compared to the pristine counterpart (Δ*E* = 0.27 V vs. 0.36 V). Such polarization mitigation is associated with ASA‐induced interfacial regulation, which reduces direct electrode‐electrolyte contact while maintaining favorable Li^+^ ‐interacting sites, thereby supporting more reversible (de)lithiation behavior. Notably, the modified cells demonstrate both intensified redox peak currents and improved peak symmetry, suggesting enhanced electronic conductivity and more reversible electrochemical kinetics. This reduction in polarization is consistent with the improvement in reaction kinetics and the enhancement of lithium‐ion diffusion, as observed in the previously discussed charge‐discharge results.

To examine the influence of the surface layer on Li^+^ insertion/extraction kinetics, the galvanostatic intermittent titration technique (GITT) was used to assess the Li^+^ diffusion behavior after three cycles, as shown in Figure 3e. For the NCM811 and NCM811‐ASA electrodes, the average Li^+^ diffusion coefficients during charging are calculated to be 2.977 × 10^−9^ and 4.870 × 10^−9^ cm^2^ s^−1^, respectively. During discharge, these values shift to 2.239 × 10^−9^ and 3.315 × 10^−9^ cm^2^ s^−1^. This result agrees well with the steeper CV‐derived ip‐V^0.5^ slopes and reduced redox peak separation, confirming more favorable Li^+^ insertion/extraction kinetics after ASA modification. The improved GITT response suggests that the ASA‐derived phase‐separated interphase preserves Li^+^ accessibility while stabilizing the cathode surface, thereby supporting the enhanced rate capability and cycling durability.

To further evaluate the influence of ASA on electrolyte accessibility, electrolyte contact‐angle measurements were performed on pristine NCM811 and NCM811‐ASA electrodes (Figure S13). The initial contact angle decreases from 34.3° for pristine NCM811 to 30.5° for NCM811‐ASA, indicating improved electrolyte wettability after ASA modification. This enhanced wettability can be attributed to the polar ester and nitrile groups in ASA, which increase the affinity between the electrode surface and the carbonate‐based electrolyte. The improved electrolyte wetting is beneficial for interfacial accessibility and is consistent with the reduced polarization and higher apparent Li^+^ diffusion coefficients discussed above.

To clarify the thermodynamic origin of the enhanced interfacial Li^+^ affinity, density functional theory (DFT) calculations were performed, with computational details provided in the Supporting Information. The (104) face was chosen as it represents the predominant and electrochemically active surface of layered NCM811, providing a realistic model for the electrode‐electrolyte interface [39, 40, 41]. As shown in Figure S14, the calculated Li^+^ adsorption energy on pristine NCM811 is −3.602 eV, whereas ASA‐NCM811 exhibits a significantly stronger Li^+^ adsorption with an energy of −4.283 eV. This enhancement is attributed to the polar C═O and C≡N groups in ASA, which provide additional coordination sites and regulate the local Li^+^ coordination environment [42, 43, 44]. Taken together, the ASA‐derived interphase combines improved electrolyte wettability with favorable Li^+^ affinity and coordination, creating a well‐regulated cathode‐electrolyte interface consistent with the enhanced electrochemical kinetics revealed by CV and GITT.

To gain further insights into the impact of ASA on the electrochemical kinetics of the cathode, EIS analysis was performed. Figure S15 compares the initial‐cycle impedance of NCM811 and NCM811‐ASA, showing a reduction in the latter. Figure 3f,g presents the Nyquist plots of the cells after the sixth, 50th, 100th, and 200th cycles. Enlarged views of the high‐frequency regions are provided in Figure S16 to facilitate comparison of the first semicircle associated with the surface‐film resistance. The first high‐frequency semicircle is assigned to (R_SEI_), which arises from the surface film formed at the electrode‐electrolyte interface, whereas the mid‐frequency semicircle reflects the charge‐transfer resistance (R_ct_) of the cycled cells. After 200 cycles, the R_SEI_ of the NCM811 cathode increases significantly, with a much higher growth rate (rising from 26.3 to 76.9 Ω) than that of NCM811‐ASA (increasing from 40.3 to 62.0 Ω). The presence of the ASA artificial interface film leads to a relatively high initial R_SEI_, but it inhibits the excessive reaction between the cathode and electrolyte, thereby slowing down the subsequent growth of R_SEI_. In terms of R_ct_, after 200 cycles, the R_ct_ of NCM811‐ASA is significantly lower than that of NCM811 (372.4 Ω vs. 486.7 Ω), reflecting enhanced lithium‐ion transport and a more stable conductive network within the electrode. Finally, Figure 3h and Table S1 compare the interfacial resistance of NCM811 and NCM811‐ASA at the sixth, 50th, 100th, and 200th cycles. Before 200 cycles, the CEI layer of NCM811‐ASA stabilizes with little change. In contrast, the interfacial resistance of NCM811 continues to rise as the CEI layer formed, indicating severe electrolyte‐side reactions and cathode structural degradation. Overall, the EIS results show a more stable evolution of interfacial resistance and consistently lower charge‐transfer resistance for NCM811‐ASA during extended cycling. This impedance evolution provides electrochemical evidence that the ASA‐derived interphase decouples CEI stabilization from charge‐transfer limitation: it suppresses continuous interfacial degradation while maintaining accessible Li^+^/electron transport pathways. To further examine the voltage‐dependent impedance response during operation, in situ EIS was performed during charge/discharge and analyzed using the distribution of relaxation times (DRT) method (Figure S17). The DRT profiles provide relaxation‐time‐resolved information on the interfacial polarization processes that are partially overlapped in conventional Nyquist plots [45]. For pristine NCM811, a more pronounced γ response appears over the main relaxation‐time region across both low‐ and high‐frequency ranges during charge/discharge, indicating intensified interfacial and charge‐transfer polarization. In contrast, NCM811‐ASA exhibits a weaker and smoother γ distribution over the same voltage range, suggesting that the ASA‐derived interphase mitigates voltage‐dependent impedance accumulation. This result is consistent with the cycle‐dependent EIS analysis, further supporting the role of ASA in stabilizing interfacial charge‐transfer behavior under high‐voltage operation.

### 2.4 Surface Morphology and Composition Analysis of Cathode After Cycling

**FIGURE 4:**
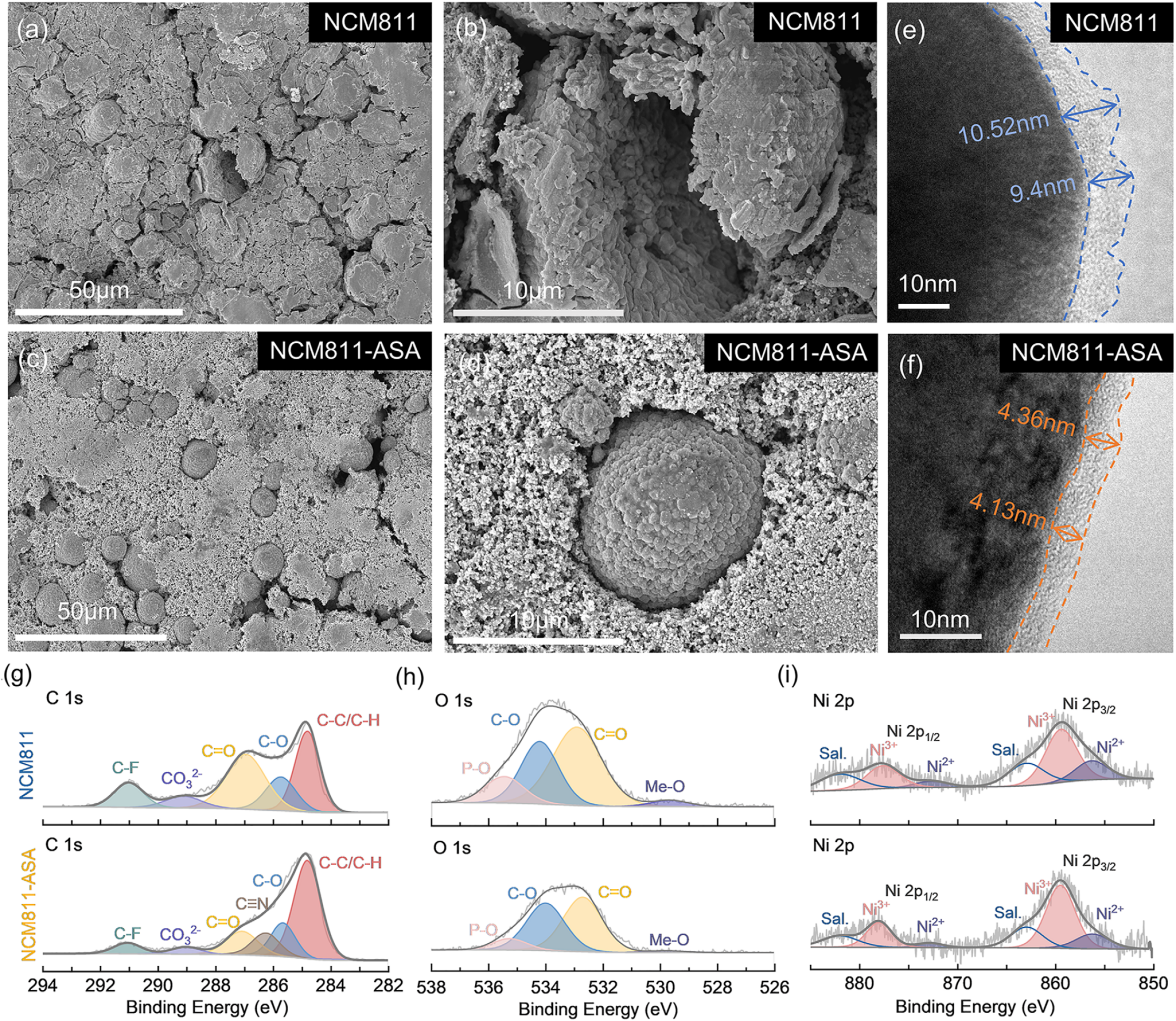
Characterization of the cycled NCM811 cathodes: SEM images of (a, b) pristine NCM811 and (c, d) NCM811‐ASA. TEM image of cycled (e) NCM811 particle and (f) NCM811‐ASA particle. XPS spectra after 200 cycles: (g) C 1s, (h) O 1s, (i) Ni 2p for both electrodes.

To further elucidate the structural and interfacial origins of the improved electrochemical performance, the surface morphology and chemical composition of the cathodes after prolonged cycling were systematically examined. After 200 cycles, pristine NCM811 particles (Figure 4a,b) exhibit severe structural destruction, with complete fracture at the center. This behavior is likely associated with stress‐driven particle cracking induced by repeated lithiation and delithiation during cycling. In addition, continuous exposure to the electrolyte can lead to corrosive attack on the NCM811 cathode, further compromising its structural integrity. In contrast, the NCM811 cathode material modified with ASA demonstrates well‐preserved spherical morphology (Figure 4c,d). This improvement is related to the elastic domains in ASA, which help relieve accumulated mechanical stress during cycling, thus reducing crack initiation and propagation. In addition, the ASA coating acts as a barrier that limits parasitic reactions between NCM811 particles and the electrolyte, helping to maintain the structural stability of the cathode [46, 47].

The thickness and morphological evolution of the CEI layers were systematically investigated through transmission electron microscopy (TEM) characterization. After 200 electrochemical cycles, the pristine NCM811 particle exhibits a newly formed CEI layer with a thickness in the range of 9.40–10.52 nm (Figure 4e), accompanied by irregular surface topography and heterogeneous morphological features. In striking contrast, the ASA‐modified NCM811 cathode demonstrates a uniform and relatively thinner CEI layer with a thickness confined to 4.13–4.36 nm (Figure 4f). This difference in CEI structure suggests the suppression of electrolyte‐cathode side reactions by ASA modification. The polymeric coating serves as an electrochemical buffer layer that mitigates excessive CEI growth and helps establish a stabilized interfacial structure, which helps maintain structural integrity during prolonged cycling.

Figure 4g–i reveals the chemical state evolution of cycled cathodes, with the deconvoluted XPS spectra reflecting surface species formed during electrochemical operation. In the C 1s spectra (Figure 4g), six distinct components are identified, corresponding to C─C/C─H (284.8 eV), C─O (285.8 eV), C≡N (286.4 eV), C═O (287.1 eV), CO_3_^2−^ (289.1 eV), and C─F (291.0 eV) [48, 49, 50]. The NCM811‐ASA electrode exhibits notably weaker C─O/C═O signals compared to pristine NCM811. These oxygenated carbon signatures, corresponding to electrolyte‐derived decomposition products, indicate suppressed interfacial parasitic reactions after polymer modification. Notably, the enhanced C─F signal in pristine NCM811 predominantly originates from PVDF binder residuals, whereas its reduced intensity in NCM811‐ASA reflects partial replacement of the binder by ASA. Meanwhile, a distinct characteristic peak of C≡N emerges.

The O 1s spectra (Figure 4h) show a pronounced decrease in the intensities of C─O (534.0 eV) and C═O (532.7 eV) bonds for NCM811‐ASA compared to pristine NCM811 [51], showing a suppression trend consistent with the C 1s results. Furthermore, the P─O signature at 534.7 eV [52] shows markedly diminished intensity in ASA‐modified cathodes, directly evidencing suppressed LiPF_6_ decomposition through the polymer stabilization effect. Additionally, the strong coordination interaction between the electronegative ‐C≡N group and Ni^3+^ is expected to reduce the formation of electrochemically inert Me─O.

As shown in Figure 4i, the binding energies of Ni 2p_3/2_ in the Ni spectra are respectively at 856.3 eV (Ni^2+^) and 859.4 eV (Ni^3+^), while those of Ni 2p_1/2_ are respectively at 872.6 eV(Ni^2+^) and 877.5 eV(Ni^3+^) [53]. Interactions between the organic functional groups and Ni species induce modifications in the surface chemical environment of the cathode [54]. The proportions of peak areas of Ni^2+^ and Ni^3+^ account for 25.2% and 74.8% in NCM811, respectively, while in NCM811‐ASA, these ratios shifted to 19.6% and 80.4%. Side reactions between the material and electrolyte reduce Ni^3+^ to Ni^2+^ [55, 56], and the similar ionic radii of Ni^2+^ and Li^+^ cause cation mixing, degrading NCM811's layered structure [48, 57]. The marked decrease in Ni^2+^ content demonstrates that the ASA‐derived artificial layer effectively inhibits the reduction of Ni^3+^ to Ni^2+^. This suppression reduces cation disorder and detrimental phase transitions, thereby improving structural stability. These results correlate with the attenuated H2→H3 peak shift in the dQ/dV curves, confirming the enhanced cycling performance of NCM811‐ASA.

As shown in Figure S18a, the contents of the electrolyte decomposition substances Li_x_PF_y_ and Li_x_PO_y_F_z_ in the P 2p spectra of NCM811‐ASA decrease, which is consistent with the reduced content of P‐O in the O 1s spectrum, indicating effective suppression of electrolyte‐side reactions. In addition, the F 1s spectra [58] (Figure S18b) and Li 1s spectra [59] (Figure S18c) show an enhanced Li‐F intensity in NCM811‐ASA compared to the pristine NCM811, suggesting a more pronounced formation of LiF. The decomposition of LiPF_6_ is illustrated in Equation S1. LiF mainly originates from the initial decomposition of LiPF_6_ (Equations S1.1 and S1.2), while a portion of free PF_5_ and its decomposition product POF_3_ react with water to form minor amounts of Li_x_PF_y_, Li_x_PO_y_F_z_, and LiF (Equations S1.3 and S1.4) [60, 61]. Notably, the increased LiF content implies a regulated interfacial decomposition behavior of PF_6_^−^, which may be associated with anion‐π interactions provided by the aromatic rings in the ASA additive, favoring LiF formation [62]. As a result, the NCM811‐ASA sample exhibits increased LiF content and the development of a robust CEI layer [63].

### 2.5 Surface Morphology and Composition Analysis of Anode After Cycling

**FIGURE 5:**
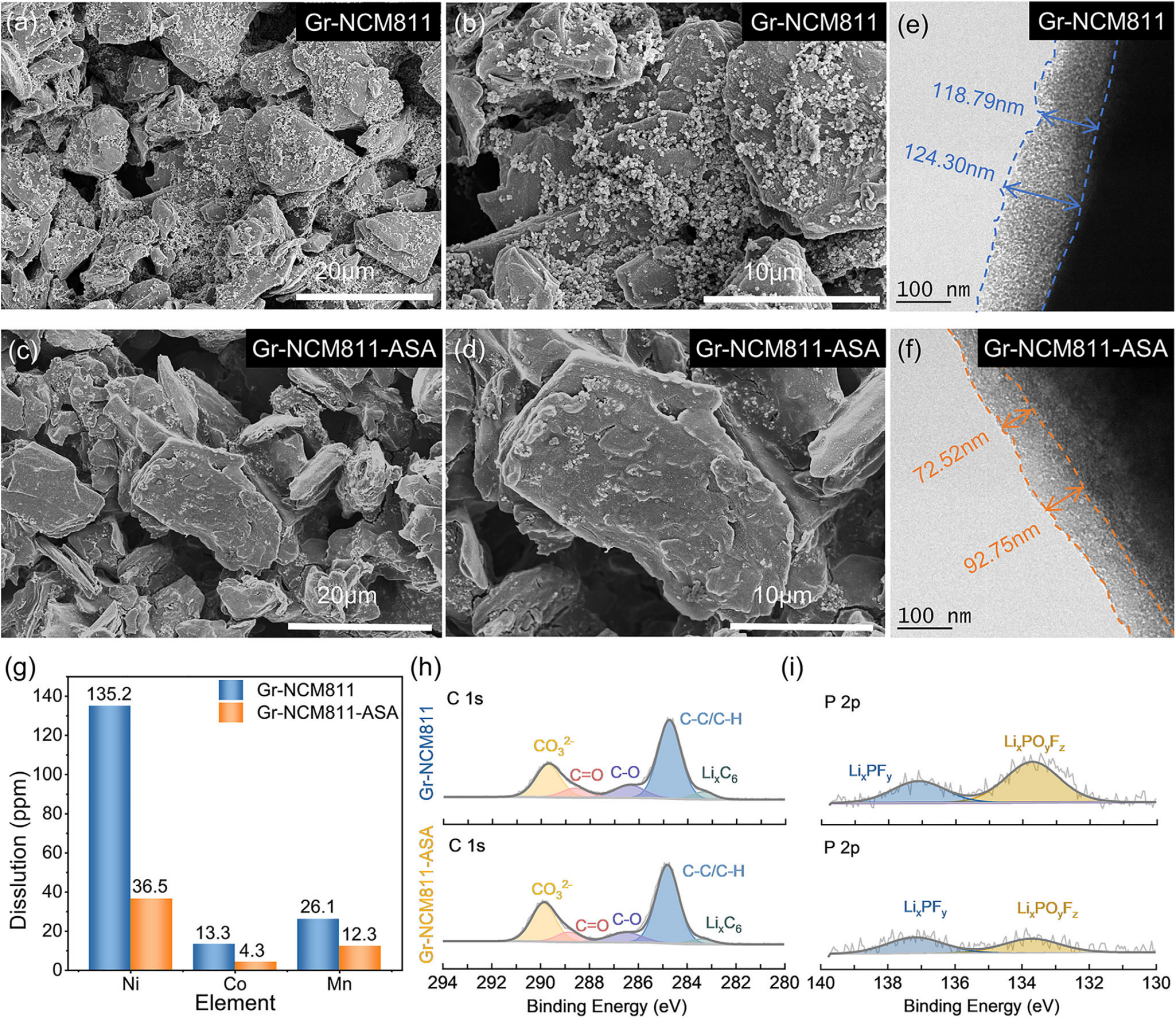
SEM images of (a, b) Gr‐NCM811 and (c, d) Gr‐NCM811‐ASA. TEM images of (e) Gr‐NCM811 and (f) Gr‐NCM811‐ASA. (g) ICP‐OES measurement of dissolved metal ion content on the graphite anodes for NCM811 and NCM811‐ASA. XPS spectra of graphite anodes harvested from NCM811||graphite and NCM811‐ASA||graphite cells after 500 cycles: (h)C 1s and (i)P 2p.

Considering the cathode‐anode crosstalk in lithium‐ion batteries, the graphite anode was further examined to determine whether the cathode‐side stabilization by ASA also mitigates downstream interfacial degradation on the anode [64, 65]. As shown in Figure 5a,b, the anode surface from pristine NCM811 batteries exhibits a rough, granular overlayer after 500 cycles, indicative of substantial SEI formation. In contrast, Figure 5c,d demonstrates that the anode surface of the NCM811‐ASA||graphite pouch cell retains a smooth topography with minimal morphological alterations, suggesting an effective suppression of continuous SEI decomposition and reformation processes. To further correlate the surface morphology with SEI composition, SEM‐EDS analysis was performed on graphite anodes (Figure S19 and Table S2). Compared with fresh graphite, the Gr‐NCM811 anode shows markedly increased F and P contents, indicating the accumulation of LiPF_6_‐derived electrolyte decomposition products on the rough surface. By contrast, the Gr‐NCM811‐ASA anode exhibits lower F and P contents, consistent with suppressed electrolyte decomposition and reduced SEI accumulation. TEM characterization was carried out to determine the thickness and morphology of the SEI layer. The TEM images were collected from representative graphite particles after surveying multiple regions. As shown in Figure 5e, a non‐uniform SEI film is observed on the cycled anode from the NCM811 cell. The SEI thickness reaches approximately 120 nm, suggesting continuous electrolyte decomposition that promotes thick SEI growth. By comparison, Figure 5f reveals a thinner, more uniform, and compact SEI layer with a thickness of 72–92 nm, implying that electrolyte decomposition on the anode side is effectively suppressed in the NCM811‐ASA cell.

To investigate transition metal dissolution during cycling, inductively coupled plasma emission spectrometry (ICP‐OES) analysis was used to quantify nickel (Ni), cobalt (Co), and manganese (Mn) elements collected from the graphite anode after 500 cycles (Figure 5g). The dissolved Ni, Co, and Mn concentrations in the NCM811‐ASA battery are approximately one‐third, one‐third, and one‐half of those in the standard NCM811 battery, respectively.

To clarify the thermodynamic origin of the reduced Ni dissolution, the Ni vacancy formation energy was further calculated. The value increases from 2.436 eV for pristine NCM811 to 4.772 eV for ASA‐modified NCM811 (Figure S20), indicating that coordination of the nitrile and ester groups with surface transition‐metal species makes Ni removal thermodynamically less favorable [66]. By limiting transition‐metal crossover and the associated catalytic decomposition of the electrolyte on graphite, ASA modification contributes to the formation of the thinner and more uniform SEI observed in the NCM811‐ASA full cell [67].

The chemical evolution of the graphite anode surface after 500 cycles in a pouch cell configuration was further examined by XPS. The C 1s spectra of cycled anodes (Figure 5h) exhibit characteristic peaks at 284.8 eV (C─C/C─H), 286.5 eV (C─O), 288 eV (C═O), and 290 eV (CO_3_^2−^) [68]. Comparatively, the graphite anode of the Gr‐NCM811‐ASA cell exhibits weaker C─O and C═O signals compared to the baseline NCM811 system, indicating suppressed electrolyte degradation at the graphite interface. A minor peak at 283 eV corresponds to residual Li_x_C_6_ in Gr‐NCM811‐ASA, suggesting enhanced electrochemical reversibility of graphite in the modified cell. The P 2p (Figure 5i) and F 1s (Figure S21a) spectra show reduced intensities of F‐ and P‐containing compounds on the graphite anode in Gr‐NCM811‐ASA, which are attributed to the decomposition of electrolyte components, including carbonates, alkyl carbonates, and carboxylates [17, 69]. Their reduced presence suggests mitigated damage to the SEI [70]. More pronounced differences are observed in Ni 2p spectra (Figure S21b): the pristine NCM811 anode shows a prominent Ni 2p3/2 peak at 857 eV, consistent with ICP‐OES‐detected nickel dissolution. In contrast, the modified cell exhibits minimal Ni signal, confirming effective suppression of transition metal migration.

**FIGURE 6:**
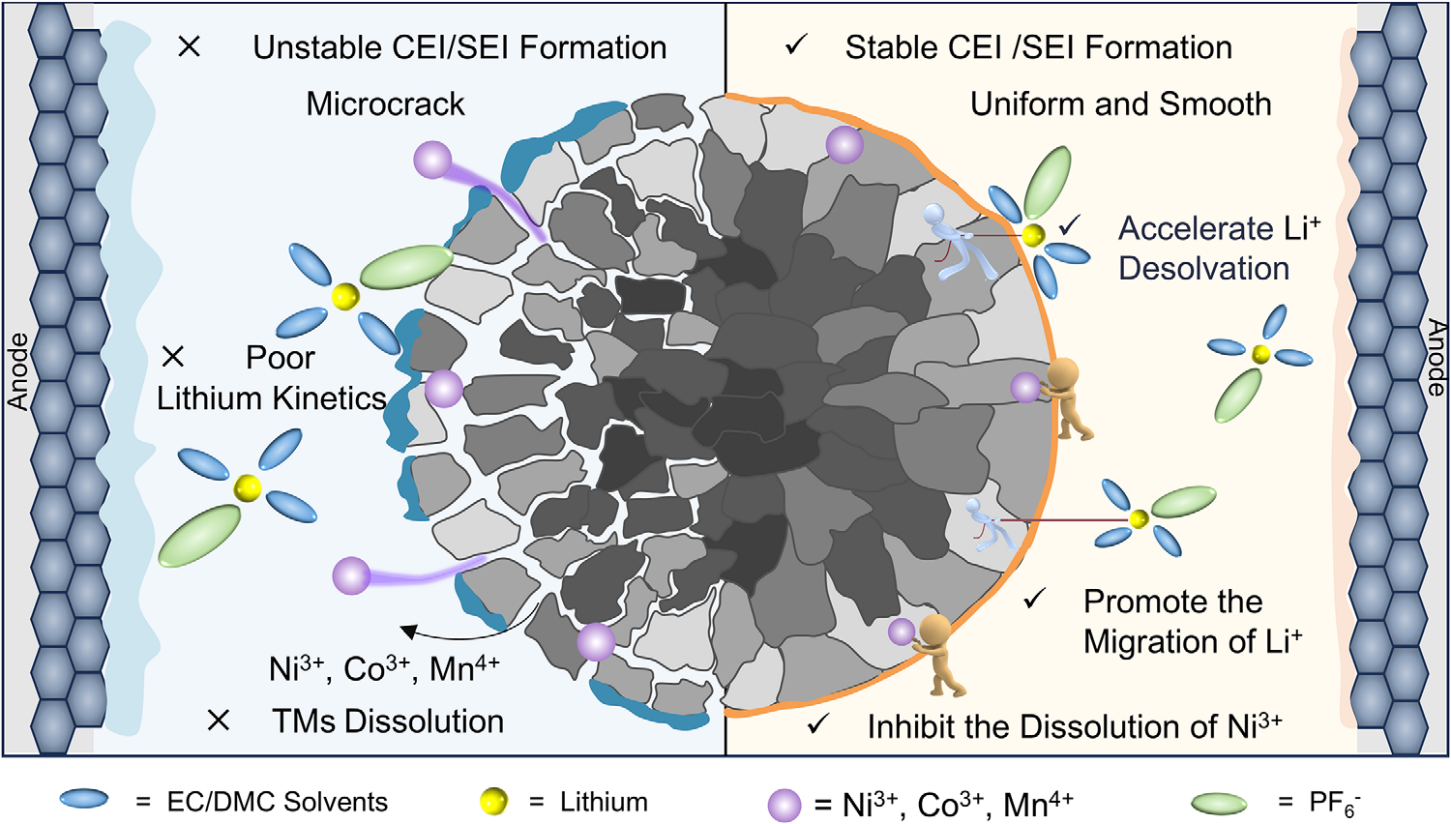
Schematic illustration of the proposed cathode‐to‐anode degradation pathway and protective mechanism of the ASA‐derived interphase during high‐voltage full‐cell cycling.

The post‐cycling analyses establish a cathode‐to‐anode degradation pathway, as schematically summarized in Figure 6. In the pristine NCM811 full cell, cathode cracking, unstable CEI growth, and surface degradation promote transition‐metal dissolution and subsequent deposition on the graphite anode, as supported by ICP‐OES and Ni 2p XPS results. The deposited transition‐metal species accelerate electrolyte decomposition and SEI overgrowth, resulting in a rougher graphite surface, thicker SEI, and stronger decomposition‐product signals. ASA modification interrupts this degradation pathway by stabilizing the cathode surface, suppressing transition‐metal crossover, and mitigating cathode‐induced anode degradation. Specifically, the PBA‐rich elastomeric component helps buffer volume‐change‐induced stress and suppress crack propagation, while the SAN‐rich polar/aromatic component contributes to Li^+^‐related interfacial coordination and interaction with conductive carbon. Therefore, the ASA‐derived phase‐separated interphase improves full‐cell cycling stability by coupling cathode‐side protection with reduced anode‐side interfacial degradation.

## 3 Conclusions

This study presents a facile, practically compatible NCM811 surface modification strategy using ASA as a slurry additive, a ternary copolymer with a unique core‐shell structure. The excellent performance demonstrated by the ASA additive modification is closely related to the synergistic functions of the ASA‐derived PBA‐rich and SAN‐rich domains and their mosaic distribution within the electrode. The PBA‐rich domains provide mechanical compliance to buffer mechanical stress, whereas the SAN‐rich domains, containing nitrile and styrene groups, contribute to interfacial compatibility, Li^+^ transport, and interaction with conductive carbon black. At an optimized loading of 2.5 wt.%, ASA forms a conformal polymer‐derived interphase comprising phase‐separated, mosaic‐like PBA‐rich/SAN‐rich domains on the NCM811 surface, effectively alleviating interfacial degradation and stress accumulation. In full pouch cells, the capacity retention is increased by 43% (78.7% vs. 55.2%) with ASA modification, while maintaining excellent rate capability. Electrochemical analyses show reduced polarization and lower charge‐transfer resistance, which are consistent with facilitated Li^+^ transport and the preservation of charge‐transport pathways enabled by the complementary roles of the SAN‐rich and PBA‐rich domains in the mosaic interphase. In addition, SEM, TEM, and XPS characterizations confirm suppressed particle cracking and optimized surface chemistry during long‐term cycling, which is attributed to the stress regulation ability of the PBA‐rich domains and the suppression of excessive electrolyte decomposition by the ASA‐derived interphase. This slurry additive approach fully leverages the structural organization and functional complementarity of the ASA‐derived domains. It offers a scalable and universally potentially generalizable approach to enhance the voltage durability of nickel‐rich layered oxide cathodes for lithium‐ion batteries. Overall, this work highlights a slurry‐compatible functional‐interphase design principle in which mechanical stress dissipation and charge transport are spatially decoupled but electrochemically integrated.

## Author Contributions

**Jiamin Duan**: writing – original draft, visualization, formal analysis, data curation, software, validation. **Xingchen Liu**: writing – original draft, methodology, formal analysis, data curation, conceptualization. **Jiapei Li**: writing – review and editing, software, visualization, methodology. **Zhuijun Xu**: writing – review and editing, data curation, visualization, formal analysis. **Said Amzil**: writing – review and editing. **Baohu Wu**: writing – review and editing, data curation, formal analysis. **Yiyao Xiao**: methodology, data curation. **Wenjun Zhang**: writing – review and editing. **Tong Yu**: data curation. **Xiaohong Li**: data curation. **Wenwen Xue**: writing – review and editing. **Xiangyu Chen**: writing – review and editing. **Changwei Liu**: writing – review and editing. **Jie Gao**: writing – review and editing, supervision. **Peter Müller‐Buschbaum**: writing – review and editing, supervision. **Ya‐Jun Cheng**: writing – review and editing, supervision, methodology, funding acquisition. **Yonggao Xia**: writing – review and editing, project administration, resources, supervision.

## Conflicts of Interest

The authors declare no conflicts of interest.

## Supporting information




**Supporting File**: advs77552‐sup‐0001‐SuppMat.docx.

## Data Availability

The data that support the findings of this study are available from the corresponding author upon reasonable request.
