# Supplementary material for: Elastomeric Core‐Conductive Shell Slurry Additive Stabilizes High‐Voltage NCM811 Cathodes via Mechanical‐Electrical Dual Regulation
Source: Adv Sci (Weinh). 2026 Sep 1:e77552. Online ahead of print. doi: 10.1002/advs.77552 (PMC13533322; doi:10.1002/advs.77552)
Supplement: Supplementary file 1 [file ADVS-9999-e77552-s001.docx]

Supporting Information

Elastomeric Core-Conductive Shell Slurry Additive Stabilizes High-Voltage NCM811 Cathodes via Mechanical-Electrical Dual Regulation

Jiamin Duan^&^, Xingchen Liu^&^, Jiapei Li, Zhuijun Xu, Said Amzil, Baohu Wu, Yiyao Xiao, Wenjun Zhang, Tong Yu, Xiaohong Li, Wenwen Xue, Xiangyu Chen, Changwei Liu, Jie Gao^*^, Peter Müller-Buschbaum^*^, Ya-Jun Cheng^*^, Yonggao Xia^*^

J. Duan, X. Liu, S. Amzil, Y. Xiao, J. Gao, Y. Cheng, Y. Xia

Ningbo Institute of Materials Technology & Engineering, Chinese Academy of Sciences, 1219 Zhongguan West Rd, Ningbo 315201, Zhejiang, P. R. China

E-mail: xiayg@nimte.ac.cn (Y. Xia), chengyj@nimte.ac.cn (Y.-J Cheng), gaojie@nimte.ac.cn (J. Gao)

J. Duan, S. Amzil, J. Gao, Y. Xia

Center of Materials Science and Optoelectronics Engineering, University of Chinese Academy of Sciences, 19A Yuquan Rd, Shijingshan District, Beijing 100049, P. R. China

X. Liu

Shanghai Institute of Space Power-Sources, No. 2965, Dongchuan Road, Minhang District, Shanghai 200245, P. R. China

J. Li, W. Zhang

Department of Materials Science and Engineering & Center of Super-Diamond and Advanced Films (COSDAF), City University of Hong Kong, 83 Tat Chee Avenue, Kowloon, Hong Kong SAR, 999077, P. R. China

Z. Xu, P. Müller-Buschbaum

Technical University of Munich, TUM School of Natural Sciences, Department of Physics, Chair for Functional Materials, Garching, 85748, Germany

B. Wu

Jülich Centre for Neutron Science (JCNS) at Heinz Maier-Leibnitz Zentrum (MLZ) Forschungszentrum Jülich, Garching, Germany

T. Yu, X. Li

College of Chemistry, Chemical Engineering and Materials Science, Soochow University, Suzhou, Jiangsu, 215123, P.R. China

W. Xue, Y. Cheng

College of Renewable Energy, Hohai University, 1915 Hohai Ave, Changzhou 213200, Jiangsu, P. R. China

X. Chen, C. Liu

Easpring (Changzhou) New Material Co., Ltd., No. 155 Jincheng Avenue, Changzhou, 213200, Jiangsu Province, P. R. China

**Experimental part**

**Materials.**

The following chemicals were all utilized in their as-received state, thus obviating the requirement for supplementary purification steps. ASA ternary polymer was purchased from Shanghai Macklin Biochemical Co., Ltd. Polycrystalline-NCM811 and graphite were both purchased from NEWARE Company. Styrene-butadiene rubber emulsion (SBR), polyvinylidene fluoride (PVDF), and carboxymethyl cellulose (CMC) binders were prepared by Zeon, Solvay, and Daicel, respectively. Conductive carbon black (Super P) was purchased from Shanghai Huipu Chemical Co., Ltd. in China. N-methyl-2-pyrrolidone (NMP) was purchased from China Aladdin Reagent Co., Ltd. The volume ratio of the electrolyte solution is EC/DMC = 3:7 (containing 1 mol L^-1^ LiPF6), which was purchased from Zhangjiagang Guotai Huarong Chemical New Materials Co., Ltd.

**Details of the composition of each part inside the battery.**

1. Composition and proportion of the pristine cathode electrode (solid mass ratio):

PVDF: NMP = 1: 25

NCM811: Super P: PVDF =8: 1: 1

2. Composition and proportion of the modified cathode electrode (solid mass ratio):

ASA: NMP = 1: 25

NCM811: Super P: PVDF: ASA =80: 10: 7.5: 2.5

3. Composition and proportion of the anode electrode (solid mass ratio):

CMC: Water = 0.3: 20

Graphite: Super P: CMC: SBR = 90: 3: 3: 4

**Assembly of Batteries.**

Firstly, PVDF and ASA were separately prepared as polymer solutions in NMP with a mass ratio of 1: 25. Then, uniform cathode slurry was prepared by mixing NCM811, Super-P, ASA, and PVDF in mass ratios of 80: 10: 2: 8, 80: 10: 2.5: 7.5, and 80: 10: 3: 7, respectively (with the ternary copolymer additives constituting 2 wt.%, 2.5 wt.%, and 3 wt.% of the solid mass of the electrode). The slurry was uniformly coated onto aluminum foil, with a controlled areal density of 4.5 mg cm^-2^, followed by drying at 110 ℃ to obtain the cathode electrode. For the anode electrode, a mixture of graphite, Super-P, CMC, and SBR was cast onto copper foil and subsequently dried. The electrolyte used was a commercial 1.0 mol·L-1 LiPF_6_ solution, with EC/DMC as the solvent in a 3: 7 volume ratio. The separator was a PP-PE-PP (Celgard M825) type. Coin cells were assembled using the cathode, separator, lithium metal, spacer, spring, and electrolyte, while pouch full cells were assembled with the cathode, separator, graphite anode, and electrolyte. The graphite||NCM811 single-layer pouch full cells were paired according to an N/P ratio ranging from 1.07 to 1.12.

**Characterization methods.**

**Electrochemical Measurements:** NCM811||Li half-cells and NCM811||graphite full cells electrochemical tests were performed using a LAND battery testing system (Wuhan, China) with a voltage range of 2.8-4.5 V. Before long-term cycling, the cells were activated for 3 cycles at 0.2 C and 4 cycles at 0.5 C, followed by cycling at 1 C to evaluate their high-voltage cycling stability. Single-layer pouch cells were first cycled for 10 cycles at 0.1 C and then tested at 0.5 C within the same voltage window. Electrochemical impedance spectroscopy (EIS) was performed on cycled half-cells using a commercial impedance analyzer (Solartron Analytical 1470E). The measurements were conducted at open-circuit voltage after discharge, corresponding to the fully discharged state of the cathode. A sinusoidal AC voltage perturbation of 10 mV was applied over a frequency range from 100 kHz to 0.01 Hz. The impedance spectra were fitted using an equivalent circuit model to extract the surface interphase resistance (R_SEI_) and charge-transfer resistance (R_ct_). The distribution of relaxation times (DRT) analysis was performed based on Bayesian ridge regression, also known as Tikhonov regularization. The DRT function was discretized using piecewise linear functions or radial basis functions (RBFs).^[1]^ Cyclic voltammetry (CV) measurements were performed on NCM811||Li half-cells using a Solartron Analytical 1470E workstation to investigate the redox behavior and interfacial kinetics of the cathode. The cells were tested within a voltage range of 2.8-4.5 V. CV curves were collected at different scan rates of 0.1, 0.2, 0.4, 0.6, 0.8, 1.0, 1.2, and 1.4 mV s^-1^. Galvanostatic intermittent titration technique (GITT) measurements were carried out using NCM811||Li half-cells to investigate the Li⁺ diffusion behavior. Before the GITT test, the cells were activated for 3 cycles at 0.2 C. During the GITT measurement, the cells were charged at 0.2 C for 10 min, followed by a relaxation period of 60 min to reach a quasi-equilibrium state. This charge-rest process was repeated until the upper cut-off voltage was reached. The cells were then discharged under the same pulse-relaxation protocol to the lower cut-off voltage. The apparent Li⁺ diffusion coefficient was calculated according to Fick’s second law using the following equation^[2]^:

$D_{Li^{+}}=\frac{4}{\pi\tau}{[\frac{m_{B}V_{M}}{M_{B}A}]}^{2}{[\frac{\Delta E_{s}}{\Delta E_{t}}]}^{2}$	(1)

The variables used in the equation are as follows: m_B_ represents the mass of the active materials (g), V_M_ represents the molar volume of the electrode (20.33 cm^3^ mol^-1^ /equation unit), M_B_ represents the molecular weight of NCM811 (97.28 g mol^-1^), A is the entire area of the electrode (cm^2^), and τ is the duration of the current pulse (10 min).

**Characterization:** Electrolyte contact-angle measurement was performed using a contact-angle analyzer (OCA25, Dataphysics) to characterize pristine NCM811 and NCM811-ASA electrodes. A commercial carbonate electrolyte consisting of EC/DMC + LiPF_6_ served as the testing liquid with a droplet volume of 3 μL, and the initial contact angle was recorded right after the electrolyte droplet made contact with the electrode surface. Differential scanning calorimetry (DSC) measurements were carried out using a DSC 214 differential scanning calorimeter (NETZSCH, Germany). Atomic force microscopy (AFM) measurements were performed using a Dimension ICON scanning probe microscope (Bruker, USA). The chemical bonds and functional groups within ASA were analyzed using micro-Fourier-transform infrared (Micro-FTIR) spectroscopy with an ATR technique. All density functional theory (DFT) calculations were performed using the CP2K program package with the Quickstep module to investigate ASA-NCM811 interfacial interactions and evaluate the effects of ASA on Li^+^ adsorption and Ni-vacancy formation on the NCM811 (104) surface. Small-angle X-ray scattering (SAXS) experiments were performed using a laboratory-based SAXS/WAXS beamline KWS-X (XENOCS XEUSS 3.0 XL, Garching version) at the Jülich Centre for Neutron Science located at the Heinz Maier-Leibnitz Zentrum. The elastic modulus and hardness of the electrode films were determined via nanoindentation (KLA-G200) under controlled loads of 1.0 mN. Cold field emission scanning electron microscopy (SEM, Hitachi S4800) and transmission electron microscopy (TEM, JEOL2100) were utilized to characterize the morphology and local structure of NCM811 cathode surface with and without ASA coating, as well as to observe changes in the morphology and local structure of electrodes post-cycling. X-ray photoelectron spectroscopy (XPS) was applied to conduct compositional analyses of decomposition products on the surface of anode and cathode electrodes after cycling. Inductively coupled plasma emission spectrometry (ICP-OES) was employed to detect the dissolution of transition metals at the graphite anode.

**Computation Details**

All density functional theory (DFT) calculations were performed using CP2K program package with the Quickstep module, which employs the Gaussian and plane wave (GPW) method. ^[3,4]^ The CP2K input file was generated and the quantum-chemical post-processing analyses were performed using Multiwfn 3.8.^[5,6]^ The slab system was treated under periodic boundary conditions in the x and y directions, with a vacuum layer of 15-20 Å added along the z-axis to avoid interactions between periodic images. The electronic structure was described using the generalized gradient approximation (GGA) with the Perdew-Burke-Ernzerhof (PBE) exchange-correlation functional.^[7]^ Grimme’s DFT-D3(BJ) dispersion correction was applied to account for van der Waals interactions.^[8]^ Core electrons were represented using norm-conserving Goedecker-Teter-Hutter (GTH) pseudopotentials, and valence electrons were expanded in a DZVP-MOLOPT-SR-GTH basis set.^[9]^ A plane-wave cutoff of 400 Ry and a relative cutoff of 55 Ry were used. The DIIS (direct inversion in the iterative subspace) minimizer and full preconditioner were used to ensure efficient SCF convergence. The convergence threshold for the SCF cycles was set to 1.0×10^-5^. Geometry optimization was performed using the Broyden-Fletcher-Goldfarb-Shanno (BFGS) algorithm with convergence thresholds of 4.5×10^-4^ Ha/Bohr for maximum force, 3.0×10^-4^ Ha/Bohr for RMS force, 3.0×10^-3^ Bohr for maximum displacement, and 1.5×10^-3^ Bohr for RMS displacement. The adsorption energy was calculated using the following equation:

$$E_{ads}=E_{surface+adsorbate}-E_{surface}-E_{adsorbate}$$

where *E_surface+adsorbate_* is the total energy of the optimized surface with the adsorbate particle. *E_surface_* is the total energy of the clean, relaxed surface. *E_adsorbate_* is the energy of the isolated adsorbate particles calculated in a sufficiently large vacuum box.

**SAXS Details**

**Sample Preparation**

**ASA-NMP, PVDF-NMP, and ASA/PVDF-NMP**: 1 g of ASA, 1 g of PVDF, and a mixture of 0.25 g ASA and 0.75 g PVDF were separately dissolved in 25 g of NMP to obtain the corresponding polymer solutions, which were denoted as ASA-NMP, PVDF-NMP, and ASA/PVDF-NMP, respectively.

**ASA, PVDF, and ASA/PVDF films**: The above solutions were drop-cast onto 8 μm thick aluminum foil and dried in an oven at 60 °C for 24 h. After drying, the aluminum foil coated with the polymer was immersed in 0.5 M NaOH solution for 5 min, after which the polymer film was peeled off from the aluminum foil. The obtained films were rinsed with deionized water and air-dried to yield the corresponding ASA, PVDF, and ASA/PVDF films.

**ASA film in ele, PVDF film in ele, and ASA/PVDF film in ele**: the prepared polymer films were separately soaked in a commercial electrolyte (EC/DMC=3/7, v/v, 1 M LiPF_6_), yielding the corresponding samples.

**SAXS Test**

SAXS experiments were performed using a laboratory-based SAXS/WAXS beamline KWS-X (XENOCS XEUSS 3.0 XL, Garching version) at the Jülich Centre for Neutron Science located at the Heinz Maier-Leibnitz Zentrum. The X-ray source was a D2+ MetalJet (Excillum) with a liquid metal anode operating at 70 kV and 3.57 mA, providing Ga-Kα radiation with a wavelength of λ = 1.314 Å.

The SAXS profiles were fitted using SasView with a combination of broad-peak, Guinier-Porod, and Gaussian peak models. The broad-peak component was used to describe the overall scattering profile and the main diffuse peak associated with the characteristic short-range organization of the polymer structure. The Guinier-Porod contribution accounted for the low-q scattering from large-scale inhomogeneities or impurity-related structures. Additional Gaussian peak functions were introduced to describe other scattering signals arising from assembled structures. The peak positions, peak widths, scale factors, and Rg were refined, while physically reasonable parameter bounds were imposed during fitting.

The solutions ASA-NMP, PVDF-NMP, and ASA/PVDF-NMP, as well as ASA film in electrolyte, PVDF film in electrolyte, and ASA/PVDF film in electrolyte, were measured in glass capillaries with an inner diameter of 2 mm at room temperature. In contrast, ASA, PVDF, and ASA/PVDF film samples were directly mounted on the sample holder for measurements.

The sample-to-detector distances were 0.12 m and 0.65 m, covering a scattering vector range of 0.015-3.7 Å^-1^, where q is defined as $q=(4\pi/\lambda)\sin(\theta)$, and 2θ is the scattering angle.

The scattering patterns were recorded with an exposure time of 600 s and repeated six times for each sample. The SAXS patterns were normalized to an absolute scale and azimuthally averaged to obtain the intensity profiles, after which the corresponding solvent background was subtracted.

**Equation**

**Equation S1.** The decomposition reactions of lithium hexafluorophosphate (LiPF_6_)

| LiPF_6_ + H_2_O → LiF + POF_3_ + 2HF | (1.1) |  | (1.1) |
| --- | --- | --- | --- |
| LiPF_6_ →LiF + PF_5_ | (1.2) |  | (1.2) |
| PF_5_ + ne^-^ + nLi^+^ → LiF + Li_x_PF_y_ | (1.3) |  | (1.3) |
| POF_3_ + ne^-^ + nLi^+^ →LiF + Li_x_PF_y_O_z_ | (1.4) |  | (1.4) |

**Table S1.** The details of the impedance fitting data in EIS results.

**Table S2.** Surface elemental compositions of Gr-Fresh, Gr-NCM811, and Gr-NCM811-ASA determined by SEM-EDS.

**Figure S1.** SEM images of (a) pristine NCM811 particle and (b) NCM811-ASA particle.

**Figure S2.** FTIR spectra of NCM811, NCM811/ASA, and ASA. NCM811-ASA shows ASA-related ester C=O and styrene-ring vibration signals at 1730 cm^-1^ and 698 cm^-1^.

**Figure S3.** XPS spectra of NCM811-Powder and NCM811-ASA-Powder. (a) C 1s and (b) N 1s spectra

**Figure S4.** Electrostatic potential (ESP) maps of six ASA fragments with different monomer sequences.

**Figure S5.** Color scale of sign(λ_2_)ρ used in the modified independent gradient model (mIGM) analysis to distinguish the nature of interfacial interactions.

Negative values represented by blue/cyan indicate attractive interactions, near-zero values shown in green correspond to weak van der Waals interactions, and positive values represented by red indicate repulsive interactions.

**Figure S6.** DSC curves of pristine ASA powder and NMP-treated/dried ASA film at a heating rate of 10 °C/min in N_2_.

**Figure S7.** SEM of (a-b) PVDF, (c-d) ASA and (e-f) ASA/PVDF films on NCM811 electrode surfaces.

**Figure S8.** SEM images of ASA films prepared under different film-forming conditions: (a, b) aluminum foil at 110 °C, serving as the common reference; (c, d) aluminum foil at 30 °C; (e, f) glass at 110 °C.

**Figure S9.** AFM nanomechanical characterization of the phase-separated ASA film. (a) Modulus map showing the selected region a and region b. (b, c) Representative force-deformation curves acquired from regions a and b, respectively.

The PBA-rich domains exhibit greater deformation and more negative force minima than the SAN-rich domains, indicating lower stiffness and stronger tip-sample adhesion. These results confirm the mechanical contrast between the two phases and support the assignment of the PBA-rich and SAN-rich domains.

**Figure S10** (a) SAXS curves of ASA powder, ASA, PVDF, and ASA/PVDF films in NMP, respectively. (b) SAXS curves of ASA, PVDF, and ASA/PVDF films.

**Figure S11.** Charge/discharge curves of (a) NCM811||graphite and (b) NCM811-ASA-2.5%||graphite pouch cells at 0.5 C for different cycles.

**Figure S12.** Overlaid dQ/dV profiles of NCM811||graphite and NCM811-ASA-2.5%||graphite pouch cells at the (a) 2nd and (b) 300th cycles.

**Figure S13.** Electrolyte contact-angle measurements of pristine NCM811 and NCM811-ASA electrodes.

**Figure S14.** DFT-calculated Li^+^ adsorption energies on the (104) surfaces of pristine NCM811 and NCM811-ASA.

**Figure S15.** Initial-cycle Nyquist plots of NCM811 and NCM811-ASA half-cells measured over a frequency range of 100 kHz-0.01 Hz, with the corresponding equivalent circuit shown in the inset.

**Figure S16.** Enlarged high-frequency regions of the Nyquist plots for (a) NCM811 and (b) NCM811-ASA half-cells after the 6th, 50th, 100th, and 200th cycles.

**Figure S17.** In situ DRT analysis of (a) NCM811 and (b) NCM811-ASA electrodes during charge/discharge.

DRT profiles derived from in situ EIS spectra of (a) NCM811 and (b) NCM811-ASA electrodes. The x-axis represents relaxation time τ, the y-axis represents cell voltage, and the color scale represents the DRT intensity γ. Compared with pristine NCM811, NCM811-ASA exhibits a weaker and smoother γ distribution over the main relaxation-time region, suggesting suppressed voltage-dependent interfacial polarization.

**Figure S18.** XPS spectra of NCM811 and NCM811-ASA electrodes after 200 cycles: (a) P 2p, (b) F 1s, and (c) Li 1s.

**Figure S19.** SEM-EDS analysis of graphite anodes before and after pouch-cell cycling. (a) Gr-Fresh, (b) Gr-NCM811, and (c) Gr-NCM811-ASA.

**Figure S20.** DFT-calculated Ni-vacancy formation energies on the (104) surfaces of pristine NCM811 and NCM811-ASA.

**Figure S21.** XPS spectra of graphite anodes harvested from NCM811||graphite and NCM811-ASA||graphite pouch cells after 500 cycles: (a) F 1s, (b) Ni 2p.

**References:**

[1]	T. H. Wan, M. Saccoccio, C. Chen, et al. “Influence of the Discretization Methods on the Distribution of Relaxation Times Deconvolution: Implementing Radial Basis Functions with DRTtools,” *Electrochemica Acta* 184 (2015): 483-499. https://doi.org/10.1016/j.electacta.2015.09.097

[2]	X. Zheng, P. Li, H. Zhu, et al., “New Insights into Understanding the Exceptional Electrochemical Performance of P2-Type Manganese-Based Layered Oxide Cathode for Sodium-Ion Batteries,” *Energy Storage Materials* 15 (2018): 257-265. https://doi.org/10.1016/j.ensm.2018.05.001

[3]	T.D. Kühne, M. Iannuzzi, M. Del Ben, et al., “CP2K: An electronic structure and molecular dynamics software package - Quickstep: Efficient and accurate electronic structure calculations,” *The Journal of Chemical Physics* 152, no. 19 (2020) 194103. https://doi.org/10.1063/5.0007045.

[4]	J. Hutter, M. Iannuzzi, F. Schiffmann, et al., “cp2k: atomistic simulations of condensed matter systems,” *WIREs Computational Molecular Science* 4, no. 1 (2014) 15-25. https://doi.org/10.1002/wcms.1159.

[5]	T. Lu, F. Chen, “Multiwfn: A multifunctional wavefunction analyzer,” *Journal of Chemical Physics* 33, no. 5 (2012) 580-592. https://doi.org/10.1002/jcc.22885.

[6]	T. Lu, “A comprehensive electron wavefunction analysis toolbox for chemists,” *Journal of Computational Chemistry* 161, no. 8 (2024) 082503. https://doi.org/10.1063/5.0216272.

[7]	J.P. Perdew, K. Burke, M. Ernzerhof, “Generalized Gradient Approximation Made Simple,” *Physical Review Letters* 77, no. 18 (1996) 3865-3868. https://doi.org/10.1103/PhysRevLett.77.3865.

[8]	S. Grimme, S. Ehrlich, L. Goerigk, “Effect of the damping function in dispersion corrected density functional theory,” *Journal of Computational Chemistry* 32, no. 7 (2011) 1456-1465. https://doi.org/10.1002/jcc.21759.

[9]	S. Goedecker, M. Teter, J. Hutter, “Separable dual-space Gaussian pseudopotentials,” *Physical Review B* 54, no. 3 (1996) 1703-1710. https://doi.org/10.1103/PhysRevB.54.1703.
